# Intermittent Maple Syrup Urine Disease Presenting With Chronic Demyelinating Polyneuropathy: A Genetically Confirmed *DBT*‐Related Case

**DOI:** 10.1111/jns.70176

**Published:** 2026-10-21

**Authors:** Gustavo Rodrigues Ferreira Gomes, Antonio Edvan Camelo Filho, Ellen Mourão Soares Lopes, Raquel Diógenes Alencar, Erlane Marques Ribeiro, Kécia Tavares de Oliveira, Paulo Ribeiro Nóbrega, André Luiz Santos Pessoa

**Affiliations:** ^1^ Division of Neurogenetics and Neuromuscular Disorders Hospital Infantil Albert Sabin Fortaleza Ceará Brazil; ^2^ Faculty of Medicine Universidade Federal do Ceará Fortaleza Ceará Brazil

**Keywords:** EMG, MSUD, peripheral neuropathy

## Abstract

**Background and Aims:**

Maple syrup urine disease (MSUD) is an inherited disorder of branched‐chain amino acid metabolism caused by deficiency of the branched‐chain α‐keto acid dehydrogenase complex. Neurological manifestations predominantly involve the central nervous system, whereas peripheral nervous system involvement has been rarely reported.

**Results:**

We report a genetically confirmed case of intermittent MSUD caused by compound heterozygous pathogenic variants in *DBT* in a female patient who developed recurrent ataxic episodes beginning in early childhood, followed by acute ascending weakness during metabolic decompensation at 12 years of age. Initially suspected to have Guillain–Barré syndrome, subsequent biochemical and molecular investigations established the diagnosis of MSUD. Five years later, despite good metabolic control and substantial functional recovery, follow‐up nerve conduction studies, and electromyography demonstrated a chronic demyelinating sensorimotor polyneuropathy. Clinical examination additionally revealed bilateral pes cavus and persistent mild distal lower‐limb weakness.

**Interpretation:**

Only a few cases of peripheral neuropathy associated with MSUD have previously been described. Our findings expand the phenotypic spectrum by documenting the longitudinal evolution from recurrent childhood neurological episodes to chronic mixed demyelinating–axonal polyneuropathy. The clinical course supports the hypothesis that repeated metabolic decompensation may contribute to cumulative peripheral nerve injury. Although the precise mechanisms remain uncertain, mitochondrial dysfunction, impaired energy metabolism, oxidative stress, and branched‐chain amino acid and α‐keto acid toxicity provide plausible explanations.

**Conclusion:**

Peripheral neuropathy should be recognized as a potential manifestation of MSUD, including intermittent phenotypes. Long‐term neurological follow‐up incorporating electrophysiological evaluation may improve recognition of peripheral nervous system involvement and contribute to a more complete characterization of the disease.

## 1 Background and Aims

Maple syrup urine disease (MSUD) is a rare autosomal recessive inborn error of metabolism characterized by impaired catabolism of the branched‐chain amino acids (BCAAs) leucine, isoleucine, and valine [1, 2]. The disorder results from deficiency of the mitochondrial branched‐chain α‐keto acid dehydrogenase (BCKDH) complex, a multienzyme system responsible for the irreversible oxidative decarboxylation of branched‐chain α‐keto acids (BCKAs), the products of BCAA transamination [2]. This complex comprises three catalytic components: E1, encoded by *BCKDHA* and *BCKDHB*; E2, encoded by *DBT*; and E3. Pathogenic variants in these genes lead to reduced enzymatic activity and accumulation of BCAAs and their corresponding ketoacids in plasma and tissues [1, 2, 3, 4, 5].

The biochemical hallmark of MSUD is the elevation of leucine, isoleucine, valine, and their respective α‐ketoacids, α‐ketoisocaproic acid (α‐KIC), α‐ketoisovaleric acid, and α‐keto‐β‐methylvaleric acid [2]. These metabolites exert neurotoxicity through multiple mechanisms. High circulating levels of leucine competitively inhibit the transport of large neutral amino acids across the blood–brain barrier, leading to an imbalance of cerebral amino acid and disruption of neurotransmitter synthesis [2, 4, 5]. In parallel, accumulation of BCKAs disrupts mitochondrial bioenergetics and redox homeostasis, contributing to energy failure and oxidative stress [2, 5, 6]. Experimental and translational data further support a dual mechanism of brain injury involving both neurotransmitter depletion and Krebs cycle dysfunction, ultimately leading to cerebral edema, demyelination, and neuronal injury [7].

Clinically, MSUD represents a spectrum of phenotypes reflecting residual BCKD activity. The classic form, accounting for the majority of cases, presents in the neonatal period with rapidly progressive encephalopathy, feeding difficulties, hypotonia, and characteristic maple syrup odor, often progressing to coma and death if untreated [1]. Neuroimaging and pathological studies demonstrate diffuse cerebral edema, hypomyelination, and white matter abnormalities, particularly in metabolically active brain regions [5, 8]. Milder phenotypes include intermediate, intermittent, and thiamine‐responsive forms, which may present later in life with episodic metabolic decompensation triggered by catabolic stress. Despite this clinical heterogeneity, metabolic instability and risk of neurological injury remain common features across all forms [1, 4, 5].

Management of MSUD is centered on reducing the accumulation of toxic metabolites and preventing catabolic states. The basis of therapy consists of lifelong dietary restriction of BCAAs combined with specialized amino acid formulas to maintain metabolic balance and support growth [1, 3]. Acute metabolic crises require rapid reduction of leucine levels and promotion of anabolism to prevent cerebral edema and neurological deterioration [1, 2]. Liver transplantation has emerged as an effective therapeutic option capable of restoring metabolic stability by providing sufficient BCKD activity, thus normalizing circulating BCAA levels and reducing the frequency of decompensation episodes [1, 2, 3]. Nonetheless, many patients develop long‐term neurocognitive and psychiatric complications despite treatment, suggesting that the current treatments may not fully prevent chronic neurotoxicity [5].

Although central nervous system involvement is classic of MSUD, peripheral nervous system manifestations have been less extensively studied and characterized. More recent evidence suggests that the metabolic disorder underlying MSUD—particularly mitochondrial dysfunction, energy deficit, and toxic metabolite accumulation—may also affect peripheral nerve integrity. Reports describing neuropathic features, including demyelinating and axonal patterns, raise the possibility that peripheral nerve involvement represents an underrecognized component of the disease spectrum [8, 9, 10].

In this context, we describe a child with genetically confirmed MSUD due to pathogenic variants in *DBT* presenting with polyneuropathy documented through nerve conduction studies and needle electromyography (NCS/EMG), expanding the phenotypic spectrum of peripheral nervous system involvement in this disorder.

## 2 Methods

This report describes a patient evaluated at a specialized neurogenetics and neuromuscular center in Brazil. Informed consent for publication was obtained from the patient and caregivers. All ancillary tests were performed as part of the patient's routine follow‐up. Genetic analysis was performed in a commercial CLIA‐certified laboratory using whole exome sequencing (WES). Genetic analysis of the parents was not possible due to financial limitations. Variants were classified according to the American College of Medical Genetics (ACMG) guidelines. All scientific content, including literature selection, data interpretation, and conclusions, were developed, critically reviewed, verified, and approved by the authors, who take full responsibility for the manuscript. AI models were used solely to check and improve language and manuscript organization.

## 3 Case Report

A 12‐year‐old female was evaluated for progressive limb weakness and episodic ataxia. She was the daughter of a nonconsanguineous couple and was born at 36 weeks of gestation without perinatal complications. Early neurodevelopment was unremarkable, with normal acquisition of motor and language milestones.

Starting at the age of 2 years, she began experiencing recurrent acute episodes of ataxia. These episodes were transient, with complete recovery between events, and neurological examination, biochemical workup, and brain imaging performed during interictal periods were unremarkable. At the age of 12, she was hospitalized due to an acute‐onset lower limb pain associated with difficulty walking, progressive ascending paralysis, NCS/EMG findings of severe symmetrical axonal sensorimotor polyneuropathy, nausea, and vomiting. Guillain–Barré syndrome (GBS) was initially suspected, but cerebrospinal fluid (CSF) analysis was normal, and the patient's symptoms improved gradually with supportive therapy. Soon after discharge, the patient was re‐hospitalized due to respiratory distress and dysphagia. During hospitalization, biochemical evaluation revealed elevated plasma leucine, valine, and isoleucine levels, raising suspicion for a disorder of branched‐chain amino acid metabolism.

Given the clinical and biochemical findings, WES was performed and identified two previously reported pathogenic variants in *DBT* in compound heterozygosity. The first variant, c.827T>G (p.Phe276Cys), results in a phenylalanine‐to‐cysteine substitution at Codon 276. The second variant, c.1018‐550A>G, is located in a noncanonical intronic region and is predicted to activate a cryptic splice site, resulting in aberrant mRNA processing. Together with the clinical and biochemical findings, these molecular results confirmed the diagnosis of intermittent MSUD.

At the most recent follow‐up, at 17 years of age, the patient remained clinically stable, with no further acute episodes of weakness or paralysis. Treatment included a restrictive protein diet and thiamine supplementation, and last amino‐acid chromatography was within normal limits. No intellectual disability was noted, and the patient performed well at school. Physical examination revealed mild muscle weakness in distal limbs, with difficulty in heel walking. She was able to ambulate independently and did not present with cerebellar signs, ataxia, or cranial nerve abnormalities at baseline. Notably, bilateral pes cavus was observed. A follow‐up NCS/EMG demonstrated a chronic, demyelinating sensorimotor polyneuropathy involving all four limbs, associated with moderate‐to‐severe secondary sensorimotor axonal degeneration in the lower limbs (see Table 1).

**TABLE 1:** Nerve conduction study findings in the patient with MSUD at the most recent follow‐up.

Study	Nerve	Site	Right			Left		
			DL (ms)	Amp	CV (m/s)	DL (ms)	Amp	CV (m/s)
Motor conduction	Median (APB)	Wrist	4.0	12.9 mV	—	3.8	10.2 mV	—
		Elbow	—	12.0 mV	**39.9**	—	8.9 mV	**45.4**
		Axilla	—	12.0 mV	**48.9**	—	NR	NR
	Ulnar (ADM)	Wrist	3.0	10.3 mV	**—**	2.9	6.9 mV	—
		Below elbow	—	10.0 mV	**45.5**	—	6.0 mV	**45.7**
		Above elbow	—	10.0 mV	**44.2**	—	4.9 mV	**48.5**
		Axilla	—	9.2 mV	**43.9**	—	NR	NR
	Fibular (TA)	Fibular head	2.6	**3.4 mV**	**—**	3.0	**3.7 mV**	—
		Popliteal fossa	—	**3.3 mV**	**39.8**	—	**3.0 mV**	**35.2**
	Fibular (EDB)	Ankle	—	**Absent**	—	—	**Absent**	—
	Tibial (AH)	Ankle	—	**Absent**	—	—	**Absent**	—
F‐waves	Ulnar	Min latency	**34.1**			**31.7**		
	Tibial	Min latency	**Absent**			**Absent**		
Sensory conduction	Median	Digit 2	2.94	28.5 μV	**47.7**	2.96	30.8 μV	**47.3**
	Ulnar	Digit 5	2.58	24.7 μV	**46.5**	2.59	32.3 μV	**46.3**
	Radial	Dorsum hand	1.69	26.4 μV	59.1	1.83	23.6 μV	54.8
	Sural	Calf	—	**Absent**	—	—	**Absent**	—
	Sup. fibular	Leg	—	**Absent**	—	—	**Absent**	—

*Note:* The bold values indicate abnormalities.

Abbreviations: ADM, abductor digiti minimi; AH, abductor hallucis; APB, abductor pollicis brevis; CMAP, compound muscle action potential; CV, conduction velocity; DL, distal latency; EDB, extensor digitorum brevis; MNCV, motor nerve conduction velocity; ms, milliseconds; mV, millivolts; N/A, nerve not tested; NR, not recorded; SNAP, sensory nerve action potential; SNCV, sensory nerve conduction velocity; TA, tibialis anterior; μV, microvolts.

## 4 Interpretation

Peripheral nervous system involvement reports in MSUD remain extremely rare. The first evidence came by a study conducted by Müller et al. in 1993, who identified subtle reductions in sural sensory conduction velocities in 3 of 10 clinically asymptomatic children with classic MSUD, suggesting that peripheral nerve dysfunction may occur even in the absence of overt neurological symptoms [8]. Kleopa et al. subsequently described a 25‐year‐old woman with classic MSUD who developed a severe acute axonal sensorimotor neuropathy following metabolic decompensation, with nerve biopsy demonstrating Wallerian degeneration [9]. Harty et al. later reported a patient with intermediate MSUD who developed an acute exacerbation of a predominantly axonal neuropathy during metabolic crisis, while serial electrophysiological studies and findings in an asymptomatic affected sibling suggested that chronic subclinical neuropathy preceded the acute presentation [10].

Our findings further expand this spectrum. Unlike the previously reported patients, our case demonstrates recurrent neurological episodes beginning in early childhood, followed by persistent mixed sensorimotor polyneuropathy with electrophysiological evidence of demyelination and secondary axonal degeneration, accompanied by chronic clinical signs such as pes cavus.

Overall, peripheral nerve involvement in MSUD may represent a continuum rather than a single clinical entity. This spectrum may range from subclinical electrophysiological abnormalities detectable only by NCS to chronic asymptomatic polyneuropathy, acute metabolic exacerbations of pre‐existing nerve dysfunction, and finally severe clinically overt polyneuropathy. Our case provides longitudinal support for this model by demonstrating persistent electrophysiological evidence of peripheral nerve involvement several years after the acute metabolic episode despite marked clinical and functional recovery. Given the unusual presence of peripheral neuropathy in MSUD, WES data were also reviewed for genes associated with hereditary peripheral neuropathies, including axonal and demyelinating forms of Charcot–Marie–Tooth disease. No pathogenic or likely pathogenic variants associated with hereditary neuropathy were identified.

Although the mechanisms of CNS injury in MSUD are relatively well established, the mechanisms of pathogenesis of peripheral neuropathy remain poorly understood. Nevertheless, several experimental and clinical observations provide biologically plausible explanations for peripheral nerve involvement. Experimental studies have demonstrated that leucine and, particularly, branched‐chain ketoacids disrupt mitochondrial bioenergetics, increase oxidative stress, and impair Krebs cycle metabolism. Together with competitive inhibition of large neutral amino acid transport across the blood–brain barrier, these mechanisms result in impaired neurotransmitter synthesis and energy deprivation within nervous tissue [2, 5, 7].

Even though these mechanisms have been primarily investigated in the CNS, there is no evidence that they are restricted to brain tissue. Peripheral axons rely heavily on oxidative phosphorylation to sustain axonal transport and membrane excitability. Consequently, chronic exposure to elevated BCAAs and BCKAs may affect especially long peripheral axons, resulting in progressive length‐dependent neuropathy. Instead of representing distinct pathological processes, the central and peripheral manifestations of MSUD may reflect different expressions of the same underlying metabolic and mitochondrial injury, although direct evidence for this mechanism in peripheral nerves is currently lacking.

An additional mechanism consideration relates to the specific molecular defect in our patient. *DBT* encodes the E2 transacylase component of the BCKD complex, a lipoate‐dependent enzyme structurally related to the pyruvate dehydrogenase complex [2]. Harty et al. proposed that accumulation of α‐ketoisocaproate may secondarily inhibit pyruvate dehydrogenase activity [9, 10], which is supported by experimental studies demonstrating impaired mitochondrial respiration and inhibition of brain energy metabolism by MSUD metabolites [11, 12]. The occurrence of episodic peripheral weakness in pyruvate dehydrogenase deficiency provides additional clinical analogy supporting this hypothesis [13].

Our patient experienced recurrent metabolic decompensations before the recognition of chronic demyelinating polyneuropathy, raising the possibility that repeated metabolic disturbances may have contributed to peripheral nerve injury. However, because plasma amino acid levels were not assessed during these episodes, a direct causal relationship cannot be established. In the long term, the development of chronic polyneuropathy and structural changes such as pes cavus indicates progression from intermittent dysfunction to chronic axonal degeneration and is compatible with the notion that repeated metabolic decompensation may lead to cumulative neuronal damage, even in different forms of MSUD.

Clinically, the recognition of peripheral neuropathy in MSUD results in important considerations. It suggests that neurological assessment in MSUD should extend beyond central manifestations to include a complete evaluation of peripheral nerve function, especially in patients with atypical motor symptoms. Interestingly, as seen in previous studies, even apparent asymptomatic patients with MSUD might present subclinical polyneuropathy, which may be a potential biomarker of cumulative neurological injury. Whether selected asymptomatic patients could benefit from electrophysiological surveillance must be further investigated.

In addition, recognizing manifestations of peripheral nervous system signs may build on the overall neurological assessment in MSUD as previous motor impairment in some patients may actually be partly due to peripheral nerve involvement rather than central nervous system dysfunction.

Lastly, it underscores the importance of strict metabolic control, as chronic exposure to elevated BCAA and BCKA levels may contribute to progressive neurological injury beyond acute encephalopathic episodes. Recognition of peripheral neuropathy as part of the neurological spectrum of MSUD may improve long‐term phenotypic characterization and provide new opportunities for monitoring disease progression and treatment response.

## Data Availability

The data that support the findings of this study are available from the corresponding author upon reasonable request.
